# Quantifying uncertainty, variability and likelihood for ordinary differential equation models

**DOI:** 10.1186/1752-0509-4-144

**Published:** 2010-10-28

**Authors:** Andrea Y Weiße, Richard H Middleton, Wilhelm Huisinga

**Affiliations:** 1 Hamilton Institute, National University of Ireland, Maynooth, Co. Kildare, Ireland; 2 Department of Mathematics & Computer Science, Freie Universität Berlin, Arnimallee 6, 14195 Berlin, Germany; 3 Centre for Systems Biology at Edinburgh, University of Edinburgh, Edinburgh EH9 3JD, UK; 4 Institute of Mathematics, University of Potsdam, Am Neuen Palais 10, D-14469 Potsdam, Germany

## Abstract

**Background:**

In many applications, ordinary differential equation (ODE) models are subject to uncertainty or variability in initial conditions and parameters. Both, uncertainty and variability can be quantified in terms of a probability density function on the state and parameter space.

**Results:**

The partial differential equation that describes the evolution of this probability density function has a form that is particularly amenable to application of the well-known method of characteristics. The value of the density at some point in time is directly accessible by the solution of the original ODE extended by a single extra dimension (for the value of the density). This leads to simple methods for studying uncertainty, variability and likelihood, with significant advantages over more traditional Monte Carlo and related approaches especially when studying regions with low probability.

**Conclusions:**

While such approaches based on the method of characteristics are common practice in other disciplines, their advantages for the study of biological systems have so far remained unrecognized. Several examples illustrate performance and accuracy of the approach and its limitations.

## Background

Ordinary differential equations (ODEs) are commonly used for modeling biological and biochemical systems. ODE models are often subject to considerable uncertainty and/or variability in both initial conditions and parameters [1-4]. Particularly in the case of nonlinear ODEs, it is essential to have efficient and accurate techniques for analyzing the effects of uncertainty and variability on the dynamical behavior. The effect of variations in the input on model behavior (output), the model sensitivity, can be analyzed in various ways. Most numerical approaches address the problem either by computing local sensitivity indices (partial derivatives of the solution with respect to the input variables) [5,6], by solving the ODE for a statistically large ensemble of random or quasi-random input values [7-9], or by approximating the functional relationship of the input and output [10-12]. When uncertainty can be narrowed down to 'small' perturbations, it is often sufficient to study its effects locally. It is, however, difficult to determine *a priori *if the uncertainty is small, and in many biological applications the assumption of small perturbations either is questionable (for example in pharmacokinetics [1]) or has been shown to be wrong [13,14].

To study those effects globally the sensitivity analysis problem can be formulated in terms of an ODE with random initial conditions. The task then is to determine the probability density function (pdf), or some features of the pdf, at a time *t *> 0. Approaches to global sensitivity analysis typically rely on Monte Carlo-type random or quasi-random approximations to the output distribution [7,8]. In such settings, information on the probability distribution is not directly accessible but encoded in the position and denseness of the sampling points. The quality of the approximation at a point will thus depend on the coverage of a surrounding region of that point, and involves some density estimation steps that might require problem specific knowledge [15]. Investigating regions of low probability with high accuracy generally requires prohibitively large numbers of sample points (for example, 100 sampling points in a region with 1/1000 = 0.1% of the total probability in expectation requires 100 000 sampling points). In [16], a global sensitivity analysis of ODEs was proposed based on recasting the problem in terms of a first-order partial differential equation (PDE) that describes the evolution of the associated pdf (this approach is also called 'stochastic sensitivity analysis' [5,6,16]). Knowledge of the entire pdf, in contrast to some specific observables (mean, variance etc.), fully characterizes the impact of uncertainty and variability. The PDE view also facilitates model assessment and parameter estimation studies, since the model likelihood naturally offers a way of assessing deviations of the model output from experimental data. The availability of the likelihood then gives access to a wide range of well-established numerical tools for model assessment and parameter estimation [15,17,18].

To numerically solve the particular class of PDEs that arise in ODEs with random initial data, finite difference schemes are commonly applied [5,6,16]. These methods typically become computationally prohibitive beyond three dimensions. Moreover, the numerical treatment of PDEs is generally less accessible to practitioners. The unscented Kalman filter (UKF) for time-continuous systems [19], originally designed for the approximate solution of the closely related Fokker-Planck equation, has also been applied to solve this PDE and to obtain sensitivity estimates of ODE models [20]. The UKF yields normal approximations of the output pdf, where the estimated mean and variance are second-order approximations of the true output mean and variance. As we illustrate in our examples, non-linear ODE models can easily give rise to strongly non-normal output distributions, even if the initial distribution is normal, such that in this case an analysis using the UKF will give misleading results.

It is however well-known that first-order PDEs can be solved using the method of characteristics [21,22]. With the method of characteristics, the pdf can be computed along trajectories of the system by solving the original ODE with an additional dimension for the density. This method thus provides a bridge from the intricate PDE description back to the ODE setting, where numerical solutions are readily accessible to practitioners. Propagating points along the trajectories of the system is a common feature of the method of characteristics, Monte Carlo methods and the unscented Kalman filter. In contrast to the other methods, however, the method of characteristics also propagates information (the value of the PDF) along the trajectories, and these values are exact up to the accuracy of the ODE solver. The additional computational costs of solving the extended ODE are negligible as the extra dimension does not necessitate additional sample/discretization points. In fact, since the pdf at a given state value is directly computable using the method of characteristics and its accuracy at a point does not depend on the denseness of sampling points in the surrounding region, considerably fewer discretization points are sufficient to obtain accurate estimates as compared to Monte Carlo-based methods [23].

Although the method of characteristics is known and widely used in other fields such as meteorology, e.g. [23], its applicability and potential has not been adequately recognized among the biological modeling community. In this article we review the formulation of ODEs with random inputs in terms of the PDE description and its solution via the method of characteristics. We demonstrate the use of the method of characteristics for sensitivity analysis as well as for likelihood-based model assessment and parameter estimation and illustrate its benefits and limitations by means of numerical case studies.

## Results and Discussion

### Methodology

#### ODEs with uncertain or variable input

Consider an ODE of the form

(1) $$\begin{array}{cc} \dot{x}=F(x), & x(0)=x_{0} \end{array}$$

with *x *∈ ℝ^*d*^. Typically, *x *belongs to some extended state space comprising the state variable *z *∈ ℝ^*n *^and the parameters *p *∈ ℝ^m ^of the system with *d *= *n *+ *m*. That is

(2) $$\begin{array}{ccc} x:=\left(\begin{array}{l} z\\ p \end{array}\right) & \text{and} & F\left(x\right):=\left(\begin{array}{l} f\left(z|p\right)\\ 0 \end{array}\right), \end{array}$$

where *f*(*z*|*p*) describes the dynamics of *z *given the parameter values *p*, which are assumed to remain constant in time. For example, *z *might denote the concentration of a molecular species of a metabolic network or signalling pathway, and *p *the associated reaction rate constants. Under the assumption that *F *: ℝ^*d *^→ ℝ^*d *^is continuously differentiable with respect to *x*, the initial value problem (1) is known to have a unique solution *x*(*t*) for *t *≥ 0 [24].

Uncertainty and variability in the model input can be modeled by assuming that *x*_0 _= *X*_0 _is a random variable with pdf *u*_0 _: ℝ^*d *^→ ℝ. Consequently, the solution {*X*_*t*_}_*t*≥0 _of the initial value problem (1) is a stochastic (Markov) process. For any *t *≥ 0, let us denote by *u*(*t*) = *u*(*t*, ·), $u:{\mathbb{R}}_{0}^{+}\times {\mathbb{R}}^{d}\to \mathbb{R}$, the pdf of the random variable *X*_*t*_, i.e., $\mathbb{P}[X_{t}\leq x]=\int_{-\infty }^{x}u(t,y)\text{d}y$. In this setting, the sensitivity of the ODE (1) at time *T *> 0 with respect to the initial density *u*_0 _amounts to computing the density *u*(*t*) at *t *= *T*. For continuously differentiable *F *and *u*, the density *u *satisfies the first-order linear PDE [25]

(3) $$\begin{array}{l} \frac{\partial }{\partial t}u=-\text{div}(F\cdot u)\\ =-\sum_{i=1}^{d}\frac{\partial }{\partial x_{i}}(F_{i}\cdot u),\;u(0,\cdot )=u_{0}, \end{array}$$

where *F_i _*denotes the *i*-th component of *F *, and div*F *is the sum of the partial derivatives of *F_i _*(equivalently, the trace of the Jacobian *DF*). Note that (3) is the Fokker-Planck equation corresponding to a stochastic differential equation with zero diffusion [26].

#### Computing the pdf along solutions of the ODE

It is well-known that first-order PDEs of the form (3) can be solved using the method of characteristics [21,22], which in our case is identical to the solution of (3) along the solution of the initial value problem (1). Define *ρ*(*t*) = *u*(*t, x*(*t*)), where *x*(·) denotes the solution of the initial value problem (1). Applying the chain rule, *ρ *obeys the ODE:

(4) $$\begin{array}{l} \dot{\rho }(t)=\frac{\partial }{\partial t}u\left(t,x(t)\right)+\sum_{i=1}^{d}\frac{\partial }{\partial x_{i}}u\left(t,x(t)\right)\cdot {\dot{x}}_{i}\\ =-\text{div}\left(F\cdot u(t))(x(t)\right)+\text{grad }u\left(t,x(t)\right)\cdot \dot{x}. \end{array}$$

Noting that $\dot{x}=F(x)$ and div(*F *· *u*(*t*))(*x*) = div*F *(*x*) · *u*(*t, x*) + grad *u*(*t, x*) · *F *(*x*), where grad $u=(\frac{\partial u}{\partial x_{1}},\ldots ,\frac{\partial u}{\partial x_{d}})$ is the gradient of *u*, and using (3) we finally obtain from (4)

(5) $$\dot{\rho }(t)=-\text{div}F\left(x(t)\right)\cdot \rho (t).$$

The PDE (3) can thus be solved pointwise for each *x*_0 _by solving the original ODE (1) together with an extra dimension for the density *ρ*, i.e.,

(6) $$\begin{array}{l} \dot{x}=F(x)\\ \dot{\rho }=-\text{div}F(x)\cdot \rho , \end{array}$$

with initial conditions *x*(0) = *x*_0 _and *ρ*(0) = *u*_0_(*x*_0_). Since *x *∈ ℝ^*d*^, the new system (6) has *d *+ 1 dimensions. The computational effort for solving the extra dimension is negligible compared to the information gained. The distribution *u*(*T*) at time *T *> 0 can be approximated by the following two steps:

**(I) **Discretize the region of interest in the state space ℝ^*d*^, resulting in discretization points *ξ*_*i*_(0), *i *= 1,...,*N*.

**(II) **For the initial values (*ξ*_*i*_(0), *u*_0_(*ξ*_*i*_(0))) ∈ ℝ^*d*+1^, *i *= 1,...*N*, solve the extended ODE (6) to compute the density *u*(*t*, *ξ*_*i*_(*t*))) at *t *= *T*.

This procedure directly yields the density values *u*(*t*, *ξ*_*i*_(*t*)) along the trajectories *ξ*_*i*_(*t*), *t *∈ [0, *T*] up to the accuracy of the ODE solver. In the subsequent examples we will further show how to modify step (I) in order to investigate the distribution on particular regions of the state and parameter space.

In comparison, Monte Carlo-based methods require density estimation subsequent to solving the ODE for the sample points:

**(i) **Sample the initial distribution *u*_0_, resulting in sampling points *ξ*_*i*_(0) ∈ ℝ^*d*^, *i *= 1,...,*N*.

**(ii) **For each sampling point *ξ*_*i *_solve the original ODE (1) to obtain *ξ*_*i *_(*t*) at *t *= *T*.

**(iii) **Estimate the density from the propagated points *ξ*_*i*_(*T*), *i *= 1,...,*N*; for example, by considering a neighborhood *B*(*x*) of a point *x *and approximating the pdf by the relative frequency, i.e., *u*(*T, x*) ≈ # {*ξ*_*i*_(*T*) ∈ *B*(*x*)}*/N*.

In contrast to the method of characteristics, the quality of the approximation *u*(*T, x*) at a *single *point *x *depends on the *total *number of sample points [15]. To obtain good estimates of the pdf in regions with low probability, Monte Carlo-based methods typically require very large sample sizes. The quality of the approximation may moreover depend on the structure of the pdf to be estimated, and might therefore necessitate problem specific knowledge. An example is given below, where the pdf is concentrated on a non-linear manifold due to fast, contracting directions.

We illustrate the advantages of the method of characteristics for sensitivity analysis of ODE models by two examples in gene expression. We will see that descriptors such as mean and variance may provide only poor information about the pdf. Our first example demonstrates the benefits of the method of characteristics in terms of an efficient computation of the pdf in regions with low probability. In the second example, the pdf contracts onto a lower-dimensional manifold of the state space, and the method of characteristics provides the density values directly along that manifold. In a third example we further show how the method can be used to compute the likelihood of an ODE model and thus facilitates comparison to experimental data. For the sake of simplicity, we choose normal initial distributions to describe state and parameter uncertainty and variability. The method of characteristics, however, provides a general strategy to compute model uncertainty/variability and likelihood for arbitrary distributions of initial values and parameters with the only restriction that the associated pdf *u *must be continuously differentiable. The choice of normal initial distributions also illustrates that the assumption of normal output distributions--underlying the UKF and least squares parameter estimation--is easily violated for non-linear ODEs, even if the initial distribution is normal.

### Examples

#### Sensitivity analysis and the impact of variability

**Figure 1:**
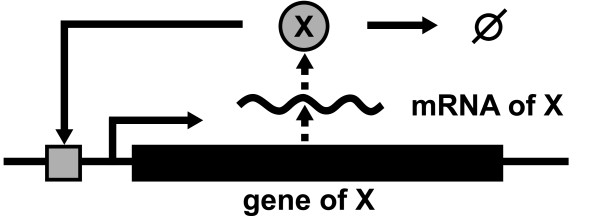
**Autoregulation**. Illustration of a protein that activates its own expression by cooperatively binding to the promoter which regulates its transcription.

**Example 1 (analyzing regions of low probability) **Consider a protein *X *activating its own expression by cooperatively binding to the promoter that positively regulates its own expression (illustrated in Figure 1). Assuming that *X *is diluted due to cell-growth with rate constant *k*_*d *_> 0, the concentration *x *of the protein is modeled by the ODE [27]

(7) $$\dot{x}=F(x)=\frac{V_{\text{max}}\cdot x^{\beta }}{K^{\beta }+x^{\beta }}-k_{d}\cdot x.$$

The first summand describes the activation of gene expression in terms of a Hill function [27], where *V*_max _> 0 denotes the maximal expression rate, *K *> 0 the protein concentration corresponding to the half-maximal expression rate, and *β *> 0 describes the cooperativity of promoter activation. A saturable synthesis was chosen to reflect a finite gene copy number encoding for *x*. The second summand describes the dilution of *x*. An initial density *u*_0 _may, for example, represent the abundance of the protein *X *in individuals of a population of cells.

**Figure 2:**
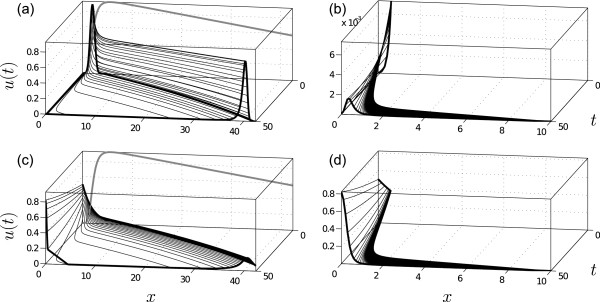
**Example 1 (autoregulated gene expression)**. (a) For an initial uniform grid on [0, 5] with grid size *h *= 0.05, the density in *t *∈ [0, 50] is computed along the trajectories. The gray line at *t *= 0 indicates the right hand side *F *(*x*) of the ODE. (b): For a final (*T *= 50) uniform grid on [0, 10] with grid size *h *= 0.1, the trajectories are computed backward in time; subsequently, forward in time, the density is computed along the trajectories. The backward-forward solution allows for computing the pdf on arbitrary sub-regions of the state space. (c) & (d) Same as (a) & (b) but with an initial exponential distribution.

Figure 2(a) shows the density *u*(*t*) in the interval *t *∈ [0, 50] by solving (6), with *F *as in (7), for a normal initial density *u*_0 _with mean *μ *= 2 (in [molvolume]) and variance *σ*^2 ^= 0.2 (in [mol2volume2]), and with parameter values *V*_max _= 1 (in [molvolume⋅time]), *K *= 2 (in [molvolume]), *k_d _*= 0.01 (in [1time]), and *β *= 4 (dimensionless).

Most of the distribution is translated linearly, since for large *x *the Hill-type activation term in (7) is approximately constant. For smaller values of *x*, however, the right hand side of (7) is strongly nonlinear. For values of *x *close to zero the dynamics are very slow, which causes the formation of a heavy tail. This is a characteristic feature of bimodality associated with positive feedback loops. Due to only few discretization points on the interval *x *∈ [0, 10] at *t *= 50 the structure of the heavy tail is only poorly resolved. In contrast to conventional Monte Carlo methods, the method of characteristics allows for a refined computation of the density on any sub-interval of interest by the following two steps:

**(I) **Discretize the sub-interval of interest, and solve the original ODE (7) for each discretization point *ξ*_*i*_(*T*), *i *= 1,...*N*, *backward *in time from *t *= *T *to 0 to obtain *ξ*_*i *_(0).

**(II) **For the initial values (*ξ*_*i *_(0), *u*_0_(*ξ*_*i*_(0))), *i *= 1,...*N*, solve (6) forward in time to compute the density along the trajectories.

**Figure 3:**
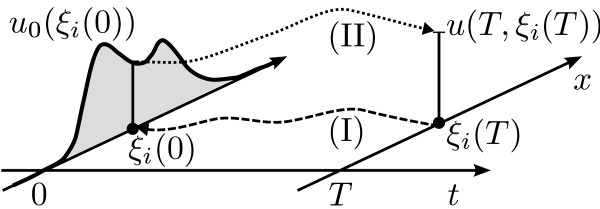
**Two-step procedure**. Illustration of the two-step procedure to compute the final pdf at specific points in space. (I) For given discretization/data points *ξ*_*i*_(*T*), *i *= 1,...*N*, the first step is to solve the original ODE backward in time (dashed arrow). (II) For the thus obtained initial values *ξ*_*i*_(0) and their initial density values *u*_0_(*ξ*_*i*_(0)), the method of characteristics is used to compute the pdf along the trajectories forward in time (dotted arrow).

The two-step procedure is illustrated in Figure 3. This way the distribution can be studied on arbitrary regions of the state and parameter space, and no subsequent normalization is required. We used the two-step procedure to obtain an improved resolution of the heavy tail (Figure 2(b)) and observe the formation of a second mode close to the origin. As an interpretation, this may imply that for this part of the population, *X *does not reach a certain threshold concentration within the given time interval (which, in turn, may be necessary to activate some other pathway). In other applications, such as toxicological risk assessment studies [28], the information may analogously be used to determine the percentage of a population that exceeds or remains below a certain toxicological threshold. To illustrate that the method of characteristics can be applied to any continuously differentiable pdf, not only normal pdfs, we repeated the above computations for an initial exponential distribution with mean *μ *= 2 (Figure 2(c) &2(d)). With standard Monte Carlo-based sensitivity approaches such localized information is difficult to obtain, when the region of interest has only low probability. The heavy tail in Figure 2(b) has a total probability of approximately 0.001, which means that in expectation only 0.1% of the Monte Carlo sampling points will lie in the interval [0, 10]. With the two-step procedure we used 100 discretization points to approximate the heavy tail. Compared to our approach, it would require 100 000 Monte Carlo sample points to expect the same coverage on the interval [0, 10], and the subsequent step of density estimation required by Monte Carlo methods further impacts the approximation quality [15].

**Figure 4:**
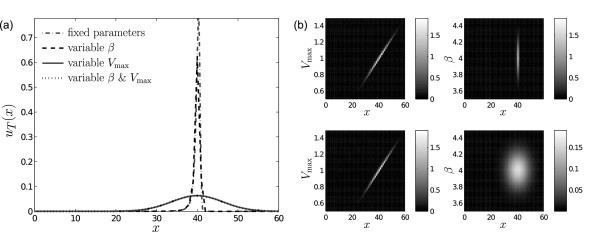
**Example 1 (autoregulated gene expression, extended state space)**. (a) Marginal distributions of protein concentration *x *at *t *= 50 for the extended variables (*x β*)' (dashed black), (*x V*_max_)' (solid gray), and (*x β V*_max_)' (dotted) compared to the distribution without parameter variability (dashed-dotted gray) computed by the two-step procedure with a final uniform grid (*x *∈ [0, 60] with *h *= 1 and *V*_max _∈ [0.5, 1.5], *β *∈ [3.5, 4.5] both with *h *= 0.01) and subsequent integration. (b) Joint distribution of (*x V*_max_)' and (*x β*)' at *t *= 50 (upper panels), and two-dimensional marginal distributions of the three-dimensional system (lower panels, note the difference in color coding by a factor of 10 between the lower right and the other panels).

Apart from variability in the initial concentration *x*, we can additionally account for variability in the parameter values. We computed the density for a state space extended according to (2) by the cooperativity *β*, by the maximal expression rate *V*_max _and by both *β *and *V*_max_, i.e., with extended state space variables (*x*, *β*)', (*x, V*_max_)' and (*x*, *β*, *V*_max_)', respectively. The initial distribution was assumed to be a joint normal distribution, where *x*(0) had mean and variance as before, and the means of *V*_max _and *β *were set to 1 and 4, respectively, each with variance 0.025. Using the above two-step procedure, we computed the densities at *T *= 50. Figure 4(a) depicts the marginal distributions of protein concentration *x *under the different scenarios of variable/uncertain parameters obtained by subsequent integration over the parameter dimensions that are not considered. For example, the marginal density in *x *is obtained from the joint density in (*x*, *β*) by

(8) u(x)=∫u(x,β)dβ.

Numerically, we discretized the above integral using the midpoint rule.

Comparison of the distributions indicates that variability in the cooperativity *β *has minor impact on the final variability of the protein concentration *x *(gray dashed-dotted vs. black dashed & solid gray vs. dotted black line). The corresponding joint distributions of (*x, V*_max_)' and (*x*, *β*)' are shown in Figure 4(b) as well as the two-dimensional marginal distributions for the three-dimensional case obtained by integrating only *β *or *V*_max_, respectively. Same as in the one-dimensional marginal distribution, it can be seen that the variability in protein concentration is mainly dominated by the variability in the maximal expression rate *V*_max_.

The numerical integration (compare eq. (8)), necessary to visualize multivariate densities for *d *≥ 3 or to compute observables such as mean and variance, is currently the computationally limiting step in the application of the method of characteristics, since it requires a regular (uniform) discretization of the state and parameter space, which becomes prohibitive in high dimensions. For low- and moderate-dimensional systems of ODEs, however, the method of characteristics provides a more efficient and equally simple alternative to conventional approaches for studying uncertainty and variability of ODE systems: The density information obtained via the method of characteristics is expectedly more accurate than estimates obtained with Monte Carlo methods [23] and considerably richer than a simple characterization of the variability/uncertainty by means of certain indicators (e.g. sensitivity indices, or variance decompositions [5-7]). It moreover facilitates further statistical analysis such as model assessment and parameter estimation, e.g., by means of information theoretical approaches [17,18], as illustrated later in Example 3 for the case of parameter estimation.

**Figure 5:**
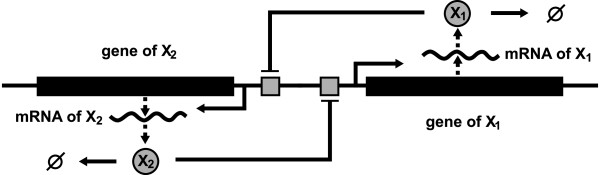
**Genetic toggle switch**. Illustration of a genetic toggle switch. The two proteins *X*_1 _and *X*_2 _mutually repress their expression by cooperatively binding to the promoter regulating the transcription of the other protein.

**Example 2 (variability along lower-dimensional manifolds) **Consider the genetic toggle switch [29], where two proteins *X*_1 _and *X*_2 _mutually repress the other protein's expression (illustrated in Figure 5). In [29], the concentrations *x*_1 _and *x*_2 _of *X*_1 _and *X*_2_, respectively, are modeled by the two-dimensional system

(9) x˙1=α11+(x2q1)β1−kd⋅x1x˙2=α21+(x1q2)β2−kd⋅x2.

The parameter *α*_1 _> 0 represents the effective expression rate of protein *X*_1_, and *β*_1 _> 0 describes the repression cooperativity of the promoter that regulates the expression of *X*_1 _by *X*_2_. Analogously, *α*_2 _and *β*_2 _describe the effective expression rate of protein *X*_2 _and its promoter's cooperativity of repression by *X*_1_, respectively. The parameter *q*_1 _(*q*_2_) corresponds to the concentration of *X*_2 _(*X*_1_) that represses the promoter activity of *X*_1 _(*X*_2_) by 50%. The parameter *k_d _*again denotes the dilution rate constant.

**Figure 6:**
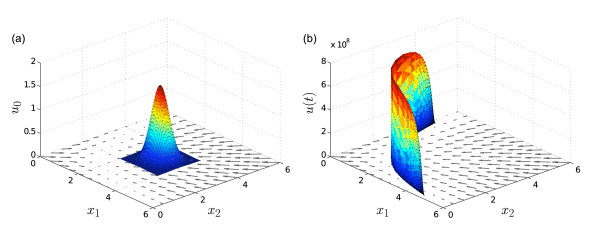
**Example 2 (genetic toggle switch)**. (a) Initial density and the vector field defined by the two-state system (9). (b) At *t *= 10 the initially normal density has contracted onto the slow manifold of the vector field and developed a steep, strongly non-normal shape with large variance along the manifold and small variance orthogonal to it.

Assume that the concentrations *x*_1 _and *x*_2 _at *t *= 0 have a joint normal distribution with mean *μ *= (3, 3)' (in [molvolume]) and covariance matrix Σ = diag{0.1, 0.1} (in [mol2volume2]). The initial density *u*_0 _is shown in Figure 6(a) along with the vector field defined by (9) with symmetric parameters *α*_1 _= *α*_2 _= *α *= 5 (in [molvolume⋅time]), *β*_1 _= *β *_2 _= *β *= 2 (dimensionless), *q*_1 _= *q*_2 _= 1 (in [molvolume]) and *k_d _*= 1 (in [1time]). We computed the density *u*(*t*) solving (6) with *F *as in (9). At *t *= 10, the majority of the probability is concentrated on the slow manifold of the vector field with large variance along the manifold (see Figure 6(b)). Such steep distributions on lower-dimensional manifolds pose problems to many other methods: Methods based on the estimation of mean and co-variance fail to describe the true shape of the distribution. Most PDE solvers have numerical problems with such steep gradients. Monte Carlo-based density estimation may yield a too coarse-grained approximation of the true density if knowledge of the manifold is not provided *a priori *; that is, to arrange the bins of a histogram or for kernel density estimation the centers of the kernel functions along the manifold. Using the method of characteristics we avoid these problems: the steep gradients do not pose any problems to the independently computed trajectories, which directly yield the density values on the attractor manifold. The method thus does not require problem specific ingenuity.

#### Deterministic models in a likelihood setting for comparison with experimental data

So far we have discussed the use of the method of characteristics to study the sensitivity of ODE models. It however also offers benefits when comparing model output with experimental data for model assessment, such as validation/falsification and selection between different models, or parameter estimation. An exact match of the deterministic model with the data is unlikely, and a quantification of the mismatch remains a critical issue. Some numerical approaches are based on verifying that the experimental data lies within regions of the state space that are reachable with certain parameter sets of the (often linearized) ODE model [30,31]. Most other approaches assess the mismatch based on root mean square deviations and select a model or parameters based on a minimization of these errors. Such least squares approaches are based on the assumption that deviations are due to additive Gaussian noise, usually assumed to be identical and independent at all points in time [32]. As we have seen in the previous examples, a normal distribution can typically not be expected for general, nonlinear ODE models, even if the initial distribution is assumed to be normal. In addition, classical least squares approaches do not allow taking into account prior information on the initial condition or on the parameter to be estimated.

Stochastic approaches offer a natural way of assessing deviations of the model output from data based on the likelihood function. Given a set of data points *D *= {*ξ*_1_,...,*ξ*_N _}, the likelihood of a model is defined as the probability that the model predicts this data. For continuous state space models *M*, the likelihood ℒ(*M*|*D*) given *D *is defined via the pdf by the product of its values at the data points

(10) $$ℒ(M|D)=\prod_{i=1}^{N}u(\xi_{i}|M),$$

where *u*(·|*M*) denotes the density function of the output distribution of the model *M*. Based on the likelihood, there are many methods available for model assessment and parameter estimation [15,18]. We can directly use the method of characteristics to efficiently compute the likelihood of an ODE model for given data points. We first consider the case that parameters are known and only initial conditions are affected by uncertainty. Given prior knowledge of this uncertainty defined by the initial density *u*_0_, and further given data points *D *= {*ξ*_1_(*T*),...,*ξ*_N_(*T*)} at a time *T *> 0, the likelihood for each single data point can be computed analogously to the two-step procedure in Example 1:

**(I) **Solve the ODE (1) for each of the *N *data points *ξ*_*i*_(*T*) ∈ ℝ^*d*^, *i *= 1,...,*N*, backward in time from *t *= *T *to 0 to obtain *ξ*_*i*_(0).

**(II) **For the initial values (*ξ*_*i*_(0), *u*_0_(*ξ*_*i *_(0))) ∈ ℝ^*d*+1^, *i *= 1,...*N*, solve the extended ODE (6) forward in time to obtain the likelihood values for each data point *u*(*T*, *ξ*_*i*_(*T*)).

In accordance with (10), the likelihood ℒ(*M*|*D*) of the model given the data is the product of the likelihood for each single data point, i.e., $ℒ(M|D)=\prod_{i=1}^{N}u\left(T,\xi_{i}(T)\right)$. As the number *N *of data points is in most cases comparatively small, the above two-step procedure is an efficient way to obtain the likelihood of an ODE model.

**Example 3 (parameter estimation) **Reconsider the ODE model (7) of autoregulated gene expression from Example 1. Assume that we want to estimate the maximal expression rate *V*_max _of the protein *X *based on an observation *ξ*(*T*) = 5 (in [molvolume]) at time *T *= 20. For sake of clarity we only consider one data point. For several data points the same procedure as described below applies, and the final likelihood is given by the product of the single-data likelihood values.

As we are interested in the likelihood of different values of *V*_max_, we consider the autoregulation model (7) extended according to (2) by *V*_max_. We apply the above two-step procedure to a representative ensemble {*υ*_1_,...,*υ*_*N*_} of values of *V*_max_. For each pair (*ξ*(*T*), *υ*_*i*_) the backward-solution yields a different value (*ξ*(0), *υ*_*i*_). Given prior knowledge in terms of a joint pdf *u*_0 _for *x*_0 _and *V*_max_, the forward-solution of (6) with initial conditions ((*ξ*_*i*_(0), *υ*_*i*_), *u*_0_(*ξ*_*i*_(0), *υ*_*i*_)) then yields the likelihood values *u*(*T*, (*ξ*(*T*), *υ*_i_)) associated with each *v*_i_, *i *= 1,...,*N*.

**Figure 7:**
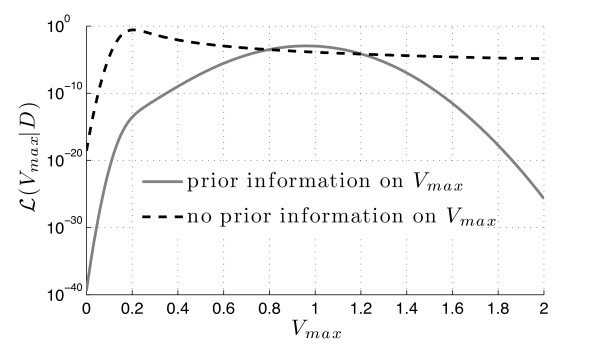
**Example 3 (parameter estimation)**. For a fictional data point *ξ *= 5 at *T *= 20 we computed the likelihood of different values of *V*_max _∈ [0, 2] (uniform discretization points with grid size *h *= 0.05) applying the two-step procedure as illustrated in Figure 3 to the autoregulation system (7) from Example 1 extended by *V*_max_. Shown are two different likelihood functions corresponding to (a) prior information imposed on both *x*_0 _and *V*_max _by means of a joint normal distribution (solid gray line), and (b) no prior information on *V*_max _in terms of *V*_max _assumed to be uniformly distributed on [0, 2] (dashed black line).

We computed the likelihood of a set of equidistant values of *V*_max _∈ [0, 2] using the same parameter values as in Example 1 (shown in Figure 7) for two different scenarios of prior information: (a) a joint normal distribution of *x*_0 _and *V*_max _with parameters *μ *= (2, 1)' and Σ = diag{0.2, 0.01} (solid gray line), and (b) a joint distribution of *x*_0 _and *V*_max_, where *x*_0 _is normally distributed with N(2, 0.2) and *V*_max _is independently uniformly distributed on the interval [0, 2] (dashed black line). The first scenario accounts for prior knowledge of *x*_0 _and *V*_max_, where a more or less precise knowledge of *V*_max _is given (since *σ*^2^(*V*_max_) is small). Accordingly, the maximum-likelihood estimate is $V_{\max}^{*}\approx 1$ close to the prior mean of *V*_max_. In the second scenario no prior information on *V*_max _was imposed (except for its constraint within [0, 2]). The maximum-likelihood estimate of *V*_max _is therefore solely determined by the value of *V*_max _that yields the initial value closest to *μ*(*x*_0_) = 2. Since the data point *ξ*(*t *= 20) = 5 is relatively unlikely for larger *V*_max _(compare with Figure 2(a)), scenario (b) yields a smaller maximum-likelihood estimate of $V_{\max}^{*}\approx 0.2$.

## Conclusions

Studying the effects of uncertainty and variability in initial values or parameters of ODE models can be computationally intensive, since it generally involves solving the system a large number of times. The method of characteristics offers a simple yet accurate alternative to conventional approaches for small- and moderate-dimensional systems. The approach does not assume a particular shape of neither input nor output distribution, it only requires the pdf to be sufficiently smooth (continuously differentiable) and yields density values that are exact up the accuracy of the ODE solver used. Our first two examples illustrate how a precise characterization of the model uncertainty/variability can be obtained with only few trajectories. In this context we also demonstrated that the analysis can be efficiently restricted to certain sub-regions of the state/parameter space. One limitation of the two-step procedure used for the latter analysis is that for chaotic models the backward-forward solution of the ODE is ill-conditioned [23]. In such cases one may resort to approximate techniques as the UKF [20], but care must be taken that the assumption of the Kalman filter, that the distribution remains approximately normal, is satiesfied. Another limitation, currently the main one, is the need for uniform grids when dimensions are to be integrated from the final density. But we anticipate that the method of characteristics will prove useful in the context of error control for approximate solution methods of eq. (1) or (3) such as Monte Carlo or the unscented Kalman filter in higher dimensions by providing exact values of the pdf at particular points in state space. As another application we considered the comparison of model results with experimental data. For deterministic models numerical approaches typically rely on root mean squared errors to quantify deviations. Their minimization can be interpreted as the maximum-likelihood estimate based on the assumption that deviations are normally (typically independently and identically) distributed at all times. While being a simple assumption, for general nonlinear ODE models it is rarely expected to hold. In the third example we described a simple framework, where the method of characteristics was applied to maximum-likelihood parameter estimation based on a distribution that accounts for prior knowledge of parameters and initial values and for the system dynamics.

We provide MATLAB files illustrating the method of characteristics in Additional file 1.

## Authors' contributions

AYW, WH and RHM planned and performed the research, AYW performed the numerical simulations, all authors contributed to the design and the writing of the manuscript. All authors read and approved the final manuscript.

## Supplementary Material

Additional file 1 **MATLAB files illustrating the usage of the method of characteristics**. Click here for file
